# Is anterior knee pain following anterior cruciate ligament reconstruction a consideration for graft choice, and the influence of COVID: a qualitative analysis in recreational athletes

**DOI:** 10.1186/s13102-023-00630-6

**Published:** 2023-03-13

**Authors:** Anastasia Sanjevic, Evangelos Tourvas, Mark A. Cairns, Fahad Alnuaimi, John Theodoropoulos, Tim Dwyer, Jas Chahal, Darrell Ogilvie-Harris

**Affiliations:** 1 University of Toronto Orthopaedic Sports Medicine, Women’s College Hospital, Toronto, ON Canada; 2 University Hospital of Heraklion, Crete, Greece; 3 OrthoCare Reidsville, Reidsville, NC USA; 4 Annie Penn Hospital, Reidsville, NC USA; 5 University of Toronto Division of Orthopaedic Surgery Residency, Toronto, ON Canada

**Keywords:** Anterior cruciate ligament, Anterior cruciate ligament reconstruction, Sports injuries, ACL graft

## Abstract

**Background:**

We set out to investigate whether anterior knee pain following anterior cruciate ligament reconstruction has a significant effect on patients, and whether it should influence graft choice.

**Methods:**

This was a qualitative analysis of a set of recreational athletes treated at a university hospital at about 1 year following anterior cruciate ligament reconstruction surgery. Participants were interviewed by an orthopaedic fellow and resident using structured, open-ended questions. Inductive theme analysis was used to code the data.

**Results:**

There were 4 major themes: (1) Our hypothesis was that patients would be given adequate information to make an appropriate graft choice. This hypothesis was rejected. Discussion took place, but with little details or rationale for the graft choice. The predominant theme was that the surgeon made the decision, and there was a lack of reliable information for the patient to make a choice. (2) The overall theme was that most patients had no anterior knee pain, and it did not interfere with activities of daily living. (3) One theme was that patients were able to resume all sports without restriction, but in some, the anterior knee pain interfered with the more demanding activities such as impact, cutting, and pivoting. A separate theme was that fear was a major impediment to return to sports and was not related to the anterior knee pain. (4) The overriding theme was that the generalized closures associated with the COVID-19 pandemic slowed the rehabilitation process. Although virtual care was available in general, it was not particularly satisfactory. Patients indicated that they had not been able to return to the gym or to their sporting activities as a result.

**Conclusions:**

Amongst non-competitive athletes, anterior knee pain post-anterior cruciate ligament reconstruction surgery does not significantly affect activities of daily living. Although there is a minor effect on sporting activities, the inability to return to sports is related to factors such as the COVID-19 pandemic, fear, or insufficient rehabilitation, rather than anterior knee pain. Overall, anterior knee pain is not a significant factor that plays a role in determining graft choice.

**Supplementary Information:**

The online version contains supplementary material available at 10.1186/s13102-023-00630-6.

## Background

Anterior knee pain after anterior cruciate ligament (ACL) reconstruction is a common symptom, especially when a bone tendon bone (BTB) graft is used [2, 3, 7, 9, 10, 13, 16]. In comparison, the incidence of anterior knee pain is significantly lower when the ACL is reconstructed using a quadriceps or hamstrings graft [1, 18, 19]. There is no qualitative study analyzing the specific limitations caused by postoperative anterior knee pain. There are many functional and outcome scores in the literature which analyze the presence or absence of anterior knee pain (Table 1, Table 2) [1, 9, 16], but they do not indicate the patient’s response specifically to the anterior knee complaints. As such, it is difficult to determine whether anterior knee pain should be a factor in the decision-making for graft type. The primary goal of this study is to investigate how anterior knee pain after ACL reconstruction affects patients, and whether it is a decision-making factor in graft selection. Our hypothesis was that the presence of anterior knee pain would represent a significant impairment, and that it should be a future consideration for graft choice.

**Table 1:** Incidence of anterior knee pain in meta-analysis literature

Study	BTB (%)	Hamstrings (%)	Quads (%)
Ajrawat 2019	35.7–36.9	32	5.7
Xie 2015	31.6	19	–
Li 2012	41.1	21.7	–

**Table 2:** Incidence of kneeling pain in meta-analysis literature

Study	BTB (%)	Hamstrings (%)	Quads (%)
Ajrawat	37.9–40.9	36.6	7.6
Xie 2015	53.6	25.3	–
Li 2012	39.4	19.3	–

As a secondary effect, because the study was carried out during COVID-19 when local public health guidelines required virtual physician and physiotherapy visits, we were also able to determine the effect the pandemic had on rehabilitation and outcome from a patient perspective [8, 15].

## Methods

### Aim, Design and Setting of the Study

The aim of this study is to determine whether anterior knee pain post-ACL reconstruction surgery significantly affects patients, and whether it should influence graft choice. A qualitative design was used to collect and analyze data. This methodology allowed us to assess patients’ experiences and perspectives in a way that quantitative research otherwise could not answer [4]. The study took place at Women's College Hospital, Toronto, Ontario, Canada.

### Patient Population

There was a total of 35 patients with 17 patella tendon autografts, 9 hamstring autografts, and 9 quadriceps tendon autografts. There were 20 males and 15 females. The age range was 21 to 47 years (Table 3). Patients who had completed a previous University of Toronto Orthopaedic Sports Medicine trial that assessed the same patient population were identified. Those who had minimal to no compliance issues in that trial, and who had met all inclusion criteria for this study, were contacted by a member of the patient's circle of care to introduce the study to them. All participants underwent surgery between late 2019 and early 2020 and were interviewed 9–15 months after the operation. None of the participants declined or withdrew their consent to participate.

**Table 3:** Participant demographics

Age Range	Males	Females	BTB graft	Hamstrings graft	Quads graft
21–47 years	20	15	17	9	9

### Data Collection

The study was introduced to participants via telephone, by members of the patients' primary circle of care (MC, FA). The goal was to recruit and interview participants until we reached the point of data saturation, which occurred after the 35th participant was interviewed. Further interviews were no longer required as no new data and themes were being found [12]. The 10–20-minute-long interviews were conducted by an orthopaedic fellow and resident (MC, FA) over the phone and Zoom Medical, and were audio recorded. The interviews, which were pilot tested on a group of non-study patients, were structured and consisted of open-ended questions evaluating how anterior knee pain post-ACL surgery affected various aspects of the patient’s life.

### Data Analysis

Transcripts were then uploaded to Quirkos (Edinburgh, Scotland) – a software used for qualitative examination of textual data – and were assessed using inductive theme analysis. Themes and codes were shaped by and derived from the raw text data [11]. Codes were developed independently by two observers (OH, AS). Approximately 30 codes were derived from the transcriptions, which were then consolidated and made into 4 main themes.

## Results

### Key Question 1: What information was provided to the patient about graft choice and specifically, anterior knee pain?

Our hypothesis was that patients would be given adequate information to make an appropriate graft choice. The general theme is that discussion took place, but with little details or rationale for the graft choice. The predominant theme was that the surgeon made the decision. Patients felt there was little information available to them to help make an informed decision. There was no difference within the gender groups of male or female, or within the groups depending on the graft type (bone tendon bone, hamstring, quadriceps).

I think he just mentioned the hamstring and we went with that. He explained the other ones but for me it was just straight forward on the hamstring graft.—Subject 1

The hamstring was presented as the option, but it wasn’t presented as a list of options.—Subject 6

No, I think he may have told me what graft we will be doing but never talked about the options.—Subject 9

The second interesting theme was that patients did a lot of research themselves. Graft choices were offered based on what their social network groups thought was advisable. Patients noted that there was little in the way of clear guidance available to them.

OK, with my doctor, in our consultation, we talked about what I did for living and he is more of an expert in sports medicine, I mean they're fairly parallel, but he is more of a hockey guy, so he got on the phone with his friend who is more of a dance surgery specialist and ask for his recommendation—Subject 2.

There wasn't that many resources available, but I also tried to look at like academic papers to see if there is any info about the recovery time—Subject 4

We mostly talked about quadriceps tendon, and patellar tendon I believe. The surgeon recommended the quadriceps tendon as one he has had success within athletes. So, I took that information and did my own research and found there is reasonable success with both options of grafts, so I was happy to go with the surgeons’ recommendation.—Subject 12.

### Key Question 2: How does anterior knee pain affect activities of daily living?

The overall theme was that patients had little or no pain and it did not interfere with activities of daily living. They stated that they could do everything that was required, although there were some patients who were symptomatic. As such, the different graft options would make no difference in terms of patients’ activities of daily living.

At this point I wouldn't say that it's affecting my quality of life at all—Subject 3

I am happy with the outcome, doing most of the stuff with no issue—Subject 6

I'm able to do everything I want to do. I can run and I have been working up to doing some jumping—Subject 13

However, we noted different themes depending on the graft type. Patients with hamstring repairs had vague symptoms of pain around the knee, but no specific limitations.

When I'm squatting, some cracking in the knee like there's something that is breaking but it is just the sound, no pain, no discomfort—Subject 1

Stairs are fine, kneeling is not painful but weird because of numbness—Subject 3

No difficulty, but I can feel more pressure when I am kneeling down—Subject 5

Patients with bone tendon bone repairs had specific issues with kneeling and stairs and had difficulty with full extension of the knee. Although they had the symptoms, it did not interfere with any of their activities of daily living. This group of patients also had a specific issue with numbness around the scar.

The only time that I get any real pain is in full extension, like trying to press my knee into the ground. it's not like an incredible amount of pain but it's definitely noticeable in terms of doing daily exercises or doing pretty much anything on a day-to-day basis—Subject 3.

I never notice it but when I specifically go out of my way to extend my knee as much as possible, I will get some stiffness.—Subject 3

It's in the front, right where the surgical area was, basically on a line below my kneecap right in the front—Subject 3

Those with quadriceps tendon grafts had issues with muscle stiffness and symptoms from the graft site in the quadriceps, but did not have any specific issues with kneeling or stairs, or any limitations in their activities of daily living.

If I'm sitting for too long then my knee feels stiff, but I think that's pretty normal—Subject 4

It’s like a pain that is constantly there. But it fades away after a few minutes. It is not a sharp pain, occasionally it is sharp, but it does not stay there.—Subject 8

Every now and then, it is not 100%, I’d say, it’s a little creaky. Pain is right in the gap, just right of the kneecap (left knee).—Subject 10

### Key Question 3: Does anterior knee pain affect sporting activities?

Within this set of interviews there were 3 major themes.

In the first theme, patients were able to return to all their pre-injury activities. Any residual knee pain was minimal and did not limit them. This group of patients were particularly satisfied with the procedure. There were no noticeable differences in the graft types or sex of the patient.

I'm able to do everything I was able to do before the surgery and before the injury too—Subject 1

Not sure I'd be quite as good as I was before, but I think good in the long run. I don't expect any issues—Subject 3

In my view it was a raging success all the way across the board—Subject 7

In patients who had not returned to their pre-injury level of sports, the predominant issue was the lack of strength. Only occasionally did patients mention that the knee pain itself was a factor despite this being a specific question. In other words, our research would indicate that the anterior knee pain was not a significant factor in limiting sporting activities. It was a factor in limiting activities such as kneeling directly on the patella and in using stairs, but not in limitations of activity.

It has, but only because I am aware that my knee is not 100% strong, and it doesn’t have the confidence that the leg is ready for it. I would say that it has just reduced the fun factor—Subject 11.

Unable to take longer walks, limited to 4–5 km, can still bike, cannot play with kids, and own physical health as a result of pain—Subject 14

I can’t do running/sprinting and long hikes with elevation. Do not trust knee yet for explosive activities. I feels as though I need to continue strengthening. Unable to kick a ball…too much impact—Subject 15.

The third major theme was the fear of reinjury. This has been reported by other authors as a significant factor in failure to return to sports. Specifically, the fear of reinjury did not seem to be related to the anterior knee pain. There was, however, a significant concern regarding a lack of psychological assistance in recovery and return to sports.

I am able to do everything else … I can run and I have been working up to doing some jumping but I'm just really scared—Subject 2

Mental health aspect, thinking about future; will it get better or worse, will it progress, other surgeries needed?—Subject 14

### Key Question 4: Did the COVID-19 pandemic affect the results of anterior cruciate ligament reconstruction?

The overriding theme was that the societal response to the pandemic with generalized closures slowed the rehabilitation. Although virtual care was available, it was not particularly satisfactory. Patients indicated that they had not been able to return to the gym or to their sporting activities as a result. Patients stated that they had not fully tested the knee in the usual recreational activities because of the unavailability of gyms or sporting programs. It does not appear from the interviews as though anterior knee pain was a significant factor in affecting recovery during the pandemic.

Rehab it took a little longer because of COVID, so I'm still working on the muscle gain—Subject 1

I guess that (my fear) also could have been abated if we had had more follow up, but the pandemic happened, so all our follow-ups were few and far between—Subject 2

I can do everything that I want to do. However, given this COVID situation I'm not really trying to go play soccer—Subject 4

The recovery has been a long difficult time. For one reason, during the pandemic, I wasn’t going to the therapist as much as I would like, and the gym was closed. For a few months after surgery, I did some virtual therapy, but it wasn’t the same—Subject 5.

As a result of COVID, my physio appointments were quickly cancelled at probably just the wrong time—*Subject 7*

Squatting is bothersome as well. But I haven’t necessarily done the appropriate strength training because of COVID, not able to go to physio or the gym and stuff—Subject 10

Thereafter the pandemic hit, so I basically did not go and see a physiotherapist. So, I know for certain that I did not have a chance to re-train the muscles that I would have otherwise—Subject 11.

### Were there any poor outcomes?

There was one patient who had a particularly poor outcome. From the patient’s perspective, the recovery was long and arduous. It was useful to have this patient in the group as we felt this did indicate saturation with at least one unsatisfactory result.

It’s extremely debilitating. Any sort of physical activity I do now revolves around making adjustments to prevent the pain from happening. It is also the focus on physiotherapy to make it better. There is also a ton of activities and sports that I avoid in order to not make it worse—Subject 9.

I feel like there was a communication breakdown early on after surgery that set me off on a path to a poor recovery. Going into surgery I was naïve about how important physiotherapy was in your recovery—Subject 9.

## Discussion

We set out to investigate whether anterior knee pain following ACL reconstruction significantly affected a patient’s activities. Our hypothesis was that the presence of anterior knee pain would represent a significant impairment that would ultimately affect graft choice, and that interference with activities would be a major theme. This hypothesis was rejected.

Studies have shown that patients with ACL reconstruction often have anterior knee pain [1, 9, 16]. This has been shown to vary with the graft type. We set out to assess the effect of anterior knee pain on the patient and were not concerned regarding the percentage of patients who had pain, which has been subject to many quantitative analyses. To determine such effects on an individual basis, and to develop themes, qualitative analysis was the option of choice.

We found that although anterior knee pain was present as a theme, it did not significantly affect the patients’ quality of life or activities of daily living. Anterior knee pain, which affected high demand activities, was a minor theme in terms of sports. The major theme was that although there may have been some pain and discomfort, it was not a limiting factor. We did not expect this outcome based on review of the current medical literature and generally held beliefs.

The type of graft did not seem to affect the presence, absence, or effect of anterior knee pain. The theme of some residual difficulties was present with all three graft types. This implies that the presence or absence of anterior knee pain would not be a significant factor in graft choice.

We had hypothesized that patients would be given sufficient information for a fully informed consent, and that this would include an adverse outcome associated with anterior knee pain. This hypothesis was rejected. Based on our analysis, a more detailed description of the rationale for graft choice, as well as the rehabilitation procedures, would be beneficial. There was little or no discussion regarding anterior knee pain following the ACL reconstruction and how this would affect the patient’s activities. There is a generally held belief that choice is an important part of provision of healthcare. This includes such concepts as patient-centered care, shared decision-making, and informed choice [17]. Within the sphere of elective orthopaedic operations, this should be achievable. This could be done through a trusted independent web-based resource for patient education. The rehabilitation following ACL surgery requires a coordinated and collaborative approach amongst healthcare providers. This has been outlined in a recent article by Darren de Sa [6]. A similar recommendation and protocol has been recommended to manage the knee osteoarthritis risk following ACL injury [5].

A major issue was the lack of mental preparedness for the ACL surgery and rehabilitation. Many participants expressed that it was fear in particular that was keeping them from achieving their true potential in the post-operative period. Specifically, the fear of reinjury held patients back from returning to sports and attaining the same level of activity they had prior to their injury. The patients felt that although there would be significant value to seeking psychological help, this was not readily available. This may be a fruitful area for educational upgrading by rehabilitation providers [8, 15].

The COVID-19 pandemic affected patients’ access to rehabilitation and return to sports (6). There clearly was a negative effect in terms of muscle strength and return of function. The virtual care in general was not satisfactory. This is an expected outcome based on the societal restrictions in place.

Patients in this study were part of a general population presenting at a Canadian university teaching hospital. They were recreational athletes, thus making our study valid for the general population. These results may not apply to professional athletes who have significantly more access to resources than the general population. The results therefore will be particularly useful to the general orthopaedic surgeon, rather than those with clinical practices with more elite athletes.

## Conclusions

In the general population of recreational athletes, residual anterior knee pain following ACL reconstruction does not have a significant effect on activities of daily living. The effect on sporting activities is relatively minor, and the inability to return to sports is more related to other factors such as fear of reinjury or inadequate rehabilitation rather than the anterior knee pain. As such, anterior knee pain is not a significant factor in determining the type of graft.

Patients had poor availability of information to make appropriate choices for graft type. This needs to be addressed by various academic organizations.

The COVID-19 pandemic affected patients’ rehabilitation due to a lack of availability of resources. It has also affected patients’ ability to return to their pre-injury sporting activities and thoroughly test the reconstruction.

## Supplementary Information

**Additional file 1.** Transcripts of the qualitative interviews that took place 9-15 months post anterior cruciate ligament reconstruction surgery. The interviews, which were conducted by an orthopaedic fellow and resident, consisted of structured, open-ended questions that aimed to evaluate how postoperative anterior knee pain affected different areas of the patient's life.

## Data Availability

All data generated or analysed during this study are included in the Additional file 1.

## Abbreviations

ACL: Anterior cruciate ligament
BTB: Bone tendon bone
