# Supplementary material for: Is anterior knee pain following anterior cruciate ligament reconstruction a consideration for graft choice, and the influence of COVID: a qualitative analysis in recreational athletes
Source: BMC Sports Sci Med Rehabil. 2023 Mar 13;15:30. doi: 10.1186/s13102-023-00630-6 (PMC10008722; doi:10.1186/s13102-023-00630-6)
Supplement: Supplementary file 1 — Additional file 1. Transcripts of the qualitative interviews that took place 9-15 months post anterior cruciate ligament reconstruction surgery. The interviews, which were conducted by an orthopaedic fellow and resident, consisted of structured, open-ended questions that aimed to evaluate how postoperative anterior knee pain affected different areas of the patient's life. [file 13102_2023_630_MOESM1_ESM.docx]

# Subject 1 - 19Jan2021

Participant ID – Subject #1

Date of interview: January 19, 2021

Date of Surgery: February 19, 2020

Surgery Performed: Left knee ACL reconstruction with hamstring autograft


INTERVIEWER: So we are now recording OK ?

PATIENT: ok

INTERVIEWER: Can you tell me your age

PATIENT: 33

INTERVIEWER: 33. And what do you do for work?

PATIENT: I am a chef

INTERVIEWER: A chef, ok. In regards to your knee, when and how did you injure your knee?

PATIENT: It was November 2019, during a sparring session of muay Thai gym

INTERVIEWER: sparring session where sorry

PATIENT: Muay Thai gym ; I practice Muay Thai gym and during a sparring session I got kicked on the outside of my knee and I fell on the ground and when I stand up I felt a pop in the knee and from then it wasn't stable so I went to go check it out

INTERVIEWER: You mentioned sparring, any other sports Prior to injuring your knee?

PATIENT: No

INTERVIEWER: And how would you describe the level that competition?

PATIENT: very very mild, Very low competition, it was just easy sparring and a kick

INTERVIEWER: were you training for any competitions or matches or anything?

PATIENT: no, I was just training for fun

INTERVIEWER: got it, just training for exercise. All right when you saw your surgeon did you talk about the different graph types for ACL reconstruction

PATIENT: no, I think he just mentioned the hamstring And we went with that. He explained the other ones but for me it was just straight forward on the hamstring graft

INTERVIEWER: OK. Alright since you've had your surgery and you're recovered do you have any pain in your knee now ?

PATIENT: no, not really

INTERVIEWER: these questions might be a little bit more straightforward to you, as not all of them are applicable. Tell me about any difficulty you may have going up or down stairs, kneeling or squatting, standing for long periods of time at work?

PATIENT: Standing at the beginning .. right now I'm pretty much fine With everything. When I'm squatting, some cracking in the knee like there's something that is breaking but it is just the sound, no pain, no discomfort. I'm standing all day for work and I have no problems with that and I have no other problems just in the rehab it took a little longer because of COVID so the muscle gain I'm still working on the muscle gain in my calves and in my hamstrings but other than that it's pretty good

INTERVIEWER: alright so your surgery and in your recovery it doesn't affect your daily activities like cooking or cleaning or getting dressed?

PATIENT: Yes, no nothing. I'm able to do everything I was able to do before the surgery and before the injury too

INTERVIEWER: OK and it doesn't prevent you from doing your job?

PATIENT: Nope. and i exercise, actually the gym is closed So I can't practice Muay Thai, but I exercise at home -I'm squatting, I'm running, I ride the bike, I do everything

INTERVIEWER: but you feel as though you could return to your preferred training if the facilities were open?

PATIENT: Yeah 100%. If not 100% then very close to where it was before

INTERVIEWER: OK and so this doesn't prevent you from doing anything that you want to do?

PATIENT: no not at all. I was at work 21 days after the surgery so I had no pain at any point pretty much. I didn't use any medication from when I left the hospital and I started exercising the day of, or the day after surgery

INTERVIEWER: excellent

PATIENT: I followed the protocol in the papers I was given at the hospital, that you guys give me at the hospital

INTERVIEWER: yes Sir

PATIENT: and everything worked fine

INTERVIEWER: perfect - you may have touched on this already but how do you feel your quality of life Is now Following your surgery?

PATIENT: normal, like it was before surgery there is no major change

INTERVIEWER: excellent! OK is there anything else that you can think of that you want to bring up in regards to your surgery and your recovery?

PATIENT: no, not really. everything went pretty smooth and I have no problem at all

INTERVIEWER: excellent, alright, well that actually covers all of our questions and if you don't have any other questions I've completed all my questions and I have gotten all the information that I wanted to get. Is there anything else that I can answer for you right now?

PATIENT: Nope

INTERVIEWER: if I have follow up questions In regards to our conversation today would it be OK for me to contact you maybe set up another interview?

PATIENT: yes that would be no problem

INTERVIEWER: I'm gonna go ahead and stop recording now OK

PATIENT: yes

# Subject 2 - 19Jan2021

Participant ID – Subject #2

Date of interview: January 19, 2021

Date of Surgery: December 19, 2019

Surgery Performed: Left knee ACL reconstruction with patellar tendon autograft, partial lateral meniscectomy


INTERVIEWER: Alright so once again thank you for helping us here can you tell me your age please?

PATIENT: 31, 30 at the time of surgery

INTERVIEWER: OK well that's always helpful. What do you do for work?

PATIENT: professional dancer

INTERVIEWER: excellent, OK when and how did you injure your knee?

PATIENT: I was on stage during a performance And I was doing a big dance thing and I did it jump landed strangely and fell down and that's when I injured it in front of a bunch of people

INTERVIEWER: unfortunate

PATIENT: Yeah I could have imagined a better or more quiet spot to do it

INTERVIEWER: I'm sure you could. other than dance and for your profession did you Play any sports prior to injuring your knee?

PATIENT: you mean like in the recent past or do you mean throughout life?

INTERVIEWER: More so leading up to the injury , were there other things that you were doing?

PATIENT: not really other than just working out of the gym, some running not much but mostly a lot of dance specifically I was doing Highland dance that year so it was a lot of high jumping

INTERVIEWER: would you say that it was a competition or part of your profession or a little bit of both?

PATIENT: no I'm a professional dancer in musicals and so the style of dance can change frequently. So this one happened to take place in Scotland in the 1800s so I was doing a ton of Highland dancing at the time so it wasn't a competition, it was at work

INTERVIEWER: got it. Alright, when you saw the surgeon did you talk with the different types of graft options for ACL reconstruction?

PATIENT: Yep and this one was recommended

INTERVIEWER: OK and you had a patellar tendon graft is that right?

PATIENT: I believe so

INTERVIEWER: why did you decide on the graft you have?

PATIENT: I think the way that it was sort of floated to me by the surgeon was that if I wanted to get back to doing what I was doing, which was pretty intense, like dancing stunts and stuff, that this would be my best shot to do it, and then he consulted with a dance doctor surgeon who is with the National Ballet and he seemed to recommend it, so I went with that

INTERVIEWER: OK and so it was based on recommendations and also in consultation with someone who has a little bit more information in regards to the demands

PATIENT: what I remember is that we're in I think it was our first consultation, me and my doctor, are you all doing patients from the same doctor or should I say names?

INTERVIEWER: different doctors

PATIENT: OK, with my doctor, in our consultation, we talked about what I did for living and he is more of a an expert in sports medicine, I mean they're fairly parallel, but he is more of a hockey guy so he got on the phone with his friend who is more of a dance surgery specialist and ask for his recommendation

INTERVIEWER: got it, OK. now as you have recovered from your surgery are you having any pain in your knee?

PATIENT: no, not pain. Actually, there is a little bit of numbness on the skin outside of the knee maybe like a cubic centimeter

INTERVIEWER: understood it's not uncommon

PATIENT: No it's not, and that's another thing that it would have been cool if, I mean, I only sort of found out it was common because my girlfriend’s cousin is a surgeon and so they told me it was normal and also we wouldn't have known if I did not ask a friend. So that would have been something good to know because I was nervous that I …

INTERVIEWER: That something had gone wrong?

PATIENT: Yeah, and I guess that also could have been abated if we had had more follow up, but the pandemic happened so all our followups were few and far between, and by phone

INTERVIEWER: yeah, things got tricky there no question

PATIENT: Yeah but I'm so grateful I got in for surgery before everything shut down

INTERVIEWER: what was the date of your surgery do you remember?

PATIENT: December 19th

INTERVIEWER: that numbness, does it cause you any concerns with you at this point or interfere with anything you want to do?

PATIENT: Not really, it makes me a little bit nervous. I know it's common, but I I'm just worried how is my knee? Is it not recovering as well as it could or will it ever be the same as it was?

INTERVIEWER: now, in terms of your knee, do you have any difficulty going up or down stairs? any problems kneeling or squatting?

PATIENT: Nope

INTERVIEWER: OK

PATIENT: like I guess in terms of activity my main problems are sort of like mental and fear

INTERVIEWER: OK we will discuss some of that here in a second

PATIENT: yeah that's on my list

INTERVIEWER: has your recovery from surgery affected your daily life, such as cooking, cleaning, getting dressed or anything like that?

PATIENT: not after the first couple of months, definitely not now

INTERVIEWER: has it affected your ability to get back to your job?

PATIENT: yes, yes in a pretty major way, I mean just the recovery time I guess, I mean I can't go back to work

INTERVIEWER: talk to me about that, are you unable to get back to work, or because of what's going on?

PATIENT: the things that I need to do at work are dancing, like after the surgery I couldn't dance, I mean I might be able to do like it's possible I could start now but it's hard to say because everything is closed

INTERVIEWER: OK

PATIENT: but that affected my ability to go back to work in a major way

INTERVIEWER: during recovery or now?

PATIENT: during recovery, yes during recovery

INTERVIEWER: now what about once you fully recovered, have you been able to get back to work?

PATIENT: well, no, because all the theaters are shut down, but that's a different thing I guess

INTERVIEWER: yes

PATIENT: I think I'd be able to go back to work and sort of like a lower impact way right now but definitely not with all the stuff I was doing before, not yet

INTERVIEWER: at this point, do you feel you could do everything you could do prior to surgery?

PATIENT: yes, I don't feel that the way I feel like I've went back and did the exact performance I was doing I don't think I could do it

INTERVIEWER: what do you think is preventing you from doing that right now?

PATIENT: I honestly think it's mostly mental

INTERVIEWER: OK

PATIENT: because I'm able to do Everything else I want to do. I can run and I have been working up to doing some jumping but I'm just really scared

INTERVIEWER: OK

PATIENT: I don't know how, I guess I'm scared for a couple of reasons. One, I don't know my leg would be able to do what it wants did and I don't wanna find out by tearing it again because the recovery was pretty brutal and I don't wanna do that again

INTERVIEWER: OK

PATIENT: my girlfriend would leave me

INTERVIEWER: we don't want to create any greater issues for you

PATIENT: Yeah that's the greater issue

INTERVIEWER: understood. So other than return to full work, full activity as part of your job is there anything that you want to do that your knee prevents you from doing?

PATIENT: let me think about that, not really, not that I can think of other than I'm just way more cautious about everything I do now. I used to do everything, used to be rough and tumble but now I don't do that kind of stuff anymore. I don't climb stuff or do anything dangerous, I don't go skiing or sports really

INTERVIEWER: high risk type activities?

PATIENT: things Where I would I feel like I would high risk activities

INTERVIEWER: some of these activities are the ones that we see commonly

PATIENT: The ones that are like going skiing, every skier has torn their ACL like five times, like that's not for me anymore no more skiing. I'm not going to be playing soccer anytime soon if ever again

INTERVIEWER: understood, otherwise how has this affected your quality of life?

PATIENT: like day to day living?

INTERVIEWER: yes

PATIENT: not much. I can do normal people activities just fine. Walking around, walking up stairs, cooking, cleaning, that's all pretty fine

INTERVIEWER: OK

PATIENT: I walk the dog a lot

INTERVIEWER: excellent. in regards to knee and your recovery and surgery, is there anything you would like to discuss or bring up?

PATIENT: yes, a couple of things - one is that I wasn't prescribed laxatives with my painkillers and I feel like that was important because I didn't poop for like 4 days and I felt like I was dying. Also, the recovery guide was really really great like we had a recovery guide from our doctor that was exercises in a timeline

INTERVIEWER: good

PATIENT: but the timeline was very ambitious. It says I can do leg lifts day one after surgery and I couldn't physically do that and like later on in my follow ups, I was worried I couldn't do these exercises and the doctor was like it's totally normal that that couldn't be done on day one so if the recovery guide had asterisks Included saying this is ideal for day one but if you can't do it that's also normal because I thought that something was wrong because I couldn't do everything that the guide said I should be able to do. I was talking to my girlfriend who was the caretaker during this thing and what could be improved for caretakers in that little guide and a guide to bandaging and sanitary care of the wound or the recovery area incision point because she was really nervous about me getting an infection and didn't quite understand how she should go about taking care of it

INTERVIEWER: OK

PATIENT: and how to do the bandages again when the original bandages came off. The other thing I know that a lot of people didn't do it traumatically like I did but for those who did like a high performance athlete or people who do this for their job it would have been really cool if there had been a referral to a sports psychologist or some sort of therapist to help get through the mental part of it's because that's still the part that's holding me back the most and I feel like I could have benefited a lot from early days of having someone who knows about the mental recovery part sort of readily available to make an appointment with

INTERVIEWER: OK

PATIENT: Because when you're sitting down recovering from what could be a career ending injury you got a lot of weird thoughts and it would be cool if you could talk to someone who is an expert in this

INTERVIEWER: with your job it's obviously physically demanding in an athlete on a team would have a trainer to work with do you have anything available like that?

PATIENT: yes we have physiotherapists and stuff but we feel that they are not as equipped to handle the mental side of it hide my visual did her best but not trained as well as a sports psychologist and they're not super readily available for dancers

INTERVIEWER: understood

# Subject 3 - 21Jan2021

Participant ID – Subject #3

Date of interview: January 21, 2021

Date of Surgery: March 4, 2020

Surgery Performed: R ACL BTB, medial and lateral meniscus repair


INTERVIEWER: so now we are recording OK?

PATIENT: Ok

INTERVIEWER: can I start by asking your age?

PATIENT: I AM 28

INTERVIEWER: and what do you do for work

PATIENT: I work at a financial institution in accounting

INTERVIEWER: when and how did you injure your knee

PATIENT: it would have been in 2019, March 2019, I was playing soccer and I I was pulled from behind and it kind of dug my foot into the ground and my I guess my cleat got stuck or something and I kept going forward. No impact or anything but that was enough to do it I guess

INTERVIEWER: you mentioned soccer, did you play any other sports prior to injuring your knee?

PATIENT: not recently but I used to play hockey and I do run quite a bit so I'd run like 10K a couple times a week

INTERVIEWER: how would you describe the level of competition of the sports that you participated in?

PATIENT: soccer I mean at the time that I injured it, I was just playing rec League. But when I was younger I used to play it up to the national level. hockey I would play up to the junior level and then with running I mean, like in terms of times, like I would finish under 40 minutes for 10 K

INTERVIEWER: when you saw the surgeon did you talk about the different graft types available for ACL reconstruction?

PATIENT: we did briefly, he mentioned that the one that I went with although over a large sample would probably result in the same outcome, theoretically the one that I went with would be more secure for someone that wanted to continue to play high level sports

INTERVIEWER: would you say that is why you decided to have the graft that you had?

PATIENT: ya, I mean I have somewhat of an understanding of my options, but I went with the one that was recommended, but he heat seemed to believe the patellar tendon graft would be the better option

INTERVIEWER: and ultimately the decision was left up to you?

PATIENT: I think so, yeah. If I remember correctly, they gave me their opinions and I think they asked me which one I wanted, but I'm pretty sure it was my decision in the end

INTERVIEWER: after the early part of your recovery, over the last couple of months have you had any pain in your knee?

PATIENT: the only time that I get any real pain is in full extension, like trying to press my knee into the ground. it's not like an incredible amount of pain but it's definitely noticeable in terms of doing daily exercises or doing pretty much anything on a day-to-day basis

INTERVIEWER: where would where is that pain that you experience when you fully extend your knee?

PATIENT: it's in the front, right where the surgical area was, basically on a line below my kneecap right in the front

INTERVIEWER: what does this pain mean to you?

PATIENT: it's a little bit frustrating but I know that it's a long recovery and it is likely just scar tissue that will be worked through, likely eventually go away so I can deal with it

INTERVIEWER: you mentioned when you force or when you fully extend your knee, any other time that you notice that pain?

PATIENT: no but that's only when I do it on a day to day basis. I never notice it but when I specifically go out of my way to extend my knee as much as possible I will get some stiffness. I guess what would be the best way to describe it like if you're trying to stretch and you just can't quite stretch all the way that's kind of the way it feels

INTERVIEWER: what relieves the pain

PATIENT: I guess just doing it consistently every day to try to continue to stretch it out more and more. I wouldn’t say that it necessarily feels bad, like I said, it's kind of like when you're trying to stretch and then you're like a little bit stiff and like almost feels good to be able to, obviously I would rather not have the pain but it does feel like I'm like stretching it out which feels, like I'm working towards something

INTERVIEWER: does the pain interfere with any of your day-to-day or sports related activities?

PATIENT: No, never. I do get like sometimes if I overdo it, I do also get like a little bit of stiffness on the sides almost like it feels like my knees like pressing in, like a little bit of pressure pressing it a little bit but that rarely ever happens and it's only if I've done a lot of exercise and honestly over the past couple months not really ever happening anymore

INTERVIEWER: any difficulty going up and down stairs, kneeling or squatting?

PATIENT: no issues going up or down. no issues squatting, basically I can do a squat just as deep as I ever could I do get a bit of pain if I kneel on a hard surface

INTERVIEWER: OK

PATIENT: It’s kind of a mix because the surgical area, there is a little bit of a Patch there were I still have some numbness. It kind of just feels odd to deal to kneel on a hard surface but it definitely improved and like on a softer surface like my bed or on the carpet or something I don't notice it

INTERVIEWER: any issues with day-to-day activities around the house cooking cleaning gardening or anything like that?

PATIENT: no nothing

INTERVIEWER: has it affected your ability to get back to sports?

PATIENT: given the situation right now I haven't really had the opportunity but in terms of my exercises, like my physio exercises and stuff like that I haven't had any issues and I've been told by PT that I'm doing extremely advanced exercises compared to the average person that would have an injury like this so I don't think it's, I would assume it's not really going to be an issue

INTERVIEWER: so if you had a chance to go back return to soccer or to the same level of running you were doing before, do you feel confident that you could do that?

PATIENT: at this point, I'm not sure I'd be quite as good as I was before but I think good in the long run I don't expect any issues

INTERVIEWER: would you say that the pain you're experiencing would not prevent you from going back?

PATIENT: no I don't think so

INTERVIEWER: has it affected your quality of life in any way?

PATIENT: I don't think so, not at this point. obviously the initial recovery was a little bit depressing but at this point I wouldn't say that it's affecting my quality of life at all

INTERVIEWER: is there anything else that you like to discuss with respect to the pain that you're having in your knee?

PATIENT: no, I don't think so. I think that covers pretty much everything

INTERVIEWER: what was the date of your surgery?

PATIENT: March 5th or March 3rd 2020

INTERVIEWER: alright well that answers all the questions I have. as we review the answers if there's anything else that I'd like to ask in regards to your knee would you be OK if I contacted you again

PATIENT: just shoot me an email I should be able to respond

INTERVIEWER: OK I appreciate your time, especially early in the morning like this

# Subject 4 - 19Jan2021

Participant ID – Subject # 4

Date of interview: January 19, 2021

Date of Surgery: January 20, 2020

Surgery Performed: Left ACL reconstruction with quadriceps

I: so we are now recording OK

P: alright

I: can you start by telling me your age

P: I am 27

I: what do you do for work?

P: I am a data scientist at a startup asset management company

I: when and how did you injure your knee?

P: I'm not entirely sure when I first injured it, so it's OK if I ramble a bit? I don't want to fill up your transcription. so I think it was like sometime February would have been of 2019 I was skiing and I did a like a little jump and then I felt my left knee I don't know it felt a bit strange and then I joked to my boyfriend being like oh I think I just tore my ACL because I had a lot of, well not a lot, but like a few people who I played soccer with because I played competitively growing up who like had to end their soccer careers because of torn ACL so I was very familiar with the injury. So when I was skiing I sort of thought oh maybe that just happened but my knee feels fine, so it was fine I didn't really notice that bothering me. Then Fast forward to September 2019, I was doing a lot of running because I wanted to do the Toronto half marathon and I remember I went for a long run on the Sunday and my left knee it felt kind of strange but I didn't really I don't know I've always just pushed through all any sort of pain or injuries I've ever had so I didn't really think much of it. Then a few days later, so probably I didn't give my body enough time to rest, I just played some soccer with some friends so yeah it was September 2019 I tore it and how it happened was I just went to like tackle somebody I guess and get the ball from them and then my knee I think I we collided so I fell and then as I was falling my knee hyper extended while I was in the air so there was no contact actually with the ground or anything and I remember it sounded like tearing paper and like a rip and it was pretty painful but I also remember thinking that it would be just an injury that would put me out for like 2 weeks but yeah clearly it was longer than that

I: you mentioned soccer and skiing, play any other sports prior to injuring your knee?

P: yeah I play ice hockey, I'm pretty active overall so I do a lot of cycling we have running as well but in terms of the main sports now it's soccer and hockey and then also skiing

I: OK and then how would you rate the level of competition sports?

P: soccer I like got to a very high level I played semi professionally like the farm team for the women's Whitecaps back in Vancouver and then hockey. I never played competitively but I think I probably could have but I chose soccer instead um yeah so I guess I would say like not professional but still like high level

I: Do you continue with those sports today

P: Hockey, I play more just like when the rinks are open. I go and play shinny and then with soccer I play back when we could play in before I was injured I would play I think like 2 times a week on Tuesdays and Saturdays usually and just recreationally with friends

I: when you saw the surgeon did you talk about the different graft options for ACL reconstruction

P: we did yeah I thought that was very interesting to hear but like hamstring graft, patellar tendon graft and then what I thought was interesting out of those, but his resident who I think was in like the very end of her residency at the time, she mentioned ‘have you thought about a quad graft’ and then I remember them also saying that for someone like me like I think they said under the age of 30, slim active female quad grafts often result in a better recovery than let's say like hamstring or patellar tendon so I just said OK sure. I was like let me do my own research on it which was really nice and yeah I just thought let's go for that

I: you mentioned your own research what kind of drew you to your your final conclusion on the quadriceps graft?

P: yeah well part of it was like I wanna just trust the professionals I didn't really have a prior going into it so I thought OK well if they suggest a quad graft is good then I'll go for it and what I was trying to read on line was like finding other peoples experiences with the quad graft, from what I understand is a pretty new graft especially in Canada so there wasn't that many resource is available but I also tried to look at like academic papers to see if there is any info about the recovery time after quad graphs and I really I didn't find anything that suggested that it was a bad idea

I: following your surgery and your recovery are you having any pain in your knee?

P: so I'm having one like a little bit of a set back I guess, and the doctor and my physio they all know about although I'm done with physio now yes so where the incisions were made I have some keilod and in particular like the lower end incision where I don't know what would have gone in there but it's a small one but that is a bit painful sometimes but if I massage it before I do my exercises then the pain goes away which I think is interesting and that's getting it's getting better and better with time but from via what doctor Theo has said I mean he's not very happy with it because it just it doesn't bother me really but it's doesn't look nice and then my physios have said that it's probably helped me back a little bit but again I don't really mind that much but yeah I think that's the source of the pain but it's minimal and then everywhere else feels fine OK

I: you mention it's minimal, the pain? does it interfere with anything?

P: no I wouldn't say it interferes anything

I: OK is there a specific time or specific movement when this pain occurs?

P: no, not really, I guess I feel it when I am doing certain exercises which happened to be my physio exercises like maybe if I'm doing single leg squats and I haven't warmed up enough or the scar massage then I'll feel it kind of feels like a pulling sensation, as if my muscles just aren't activating properly so then it feels a little painful

I: so you have mentioned massage but anything else that relieves the pain?

P: all I've tried is just like a bit of scar massage and that seems to work so yeah I haven't try it anything else

I: does it currently interfere with any of your day-to-day or sports related activities?

P: no, I can do everything that I want to do however given this COVID situation I'm not really trying to go play soccer. I'm not going to do that for a while so maybe I haven't had the opportunity to test things out as much as I would have if this was a normal time but I can play, I can kick a ball around, I've started playing tennis that's a new thing since lacrosse since my surgery actually I think function actually I feel great actually

I: with that in mind, do you have any difficulty going up and down stairs kneeling or squatting?

P: no not at all so I used to be able to do like pistol squats where you go all the way like your butt to the ground. I don't want to test it out yet but I feel that I could do that if I wanted too I'm sort of trying to work back to that so I feel like I'm really close to back to my old strength

I: it doesn't affect any daily activities getting dressed for cleaning the house gardening etc?

P: nothing, I mean I guess the only thing would be sometimes if I'm sitting for too long then my knee feels stiff but I think that's pretty normal and I expect that to be around for awhile

I: Has it affected your ability to return to sports knowing or understanding that you know limited activity right now due to the pandemic?

 P: so I think I feel that I've been very on track for returning to sports yeah

I: until you feel comfortable testing it for example if if something were to come available right now?

P: definitely yeah

I: Does pain affect your ability to complete your job?

P: Nope, especially since I have a desk job

I: have you been able to return to play soccer or hockey and if not, would you be able to so?

P: I have been I guess we can't play hockey right now because of COVID but the rinks are open so I've been skating a lot and trying to hold myself back one for my knee but also two because there's other people around so I probably shouldn't skate too fast and like OK scare them or get too close to them so yeah I feel that if I was to go back and play a game of hockey I think my knee would I would be tired because it's been so long but I do feel that I would be able to handle it and I would it would just take some time to get back into it with soccer I feel similarly but maybe not as confident because there's more planting and pivoting an it's it's like it's definitely a different motion than skating so I have noticed sometimes when I'm like kicking a ball around and all if I try to push it too hard I will like my knee it doesn't feel like it gives out but it just feels weak but I think that's also because I've done a lot more skating recently than I've done getting back and just soccer and then similarly like when I started playing tennis I felt that same sort of thing but as my muscles got more used to the particular pivoting motion in tennis that feeling started to go away so yeah I feel like I would maybe need like for five sessions of playing soccer again to feel comfortable

I: but your confident you could return?

P: yeah

I: do you have an interest to return?

P: definitely yeah

I: And has this pain affected your quality of life?

P: no not at all

I: is there anything else you would like to discuss with respect to your knee surgery your recovery or any of the discomfort that you're experiencing?

P: no I guess with the keloid, I've gotten keloids before like when I like got stitches for whatever the reason so I guess I could have known that was gonna happen but I don't know if that was ever necessarily discussed so I don't maybe it's not like I'm upset but it could be good if I can make a suggestion it could be good to add that to this the pre surgery discussion like if you are susceptible to keloids this could happen to your scar and then this could hold back your recovery just so you know like that sort of thing it wouldn't have changed any of the decisions I made to go forward with surgery and to go forward with getting the claw draft but it was definitely like a bit of a mystery to me why I got a key light but I think even like doctor Theo was not yeah no it doesn't not pleased and seemed surprised as well that it was like such a bad keloid and I remember like one of my recent post op appointments I think two of the residents came to take a look at it too because they're like oh that's so interesting so maybe I'm a unique case

I: fair enough, but also a good to note good for us to be more thorough with our discussion before surgery just to clarify is it the keloid that's causing you that the discomfort, is it below your kneecap or above the kneecap

P: below my kneecap so initially the one but I think everybody and by that I mean like the surgery team man my physio kinesiology I think they were most concerned about the incision where the quad graft was taken from so above my knee because that one is larger than the one below my knee I guess it's like shows my left knee below to the right yes so which at whatever that and I'm sure you know what those whatever that incision is it's so small but that's the one that's affecting me and it's only very slightly keloid at this point and still compared to the quad graft incision it's so small but yeah for some reason that's the one that's bothering me and but I also wonder if it could be that I spent a lot of time like really early on in my search or early on my recovery massaging the larger quad um like the incision from cloud craft yes so maybe I like managed to break down that scar tissue much better than I was able to breakdown the scar tissue in the one that's now affecting me I don't know

# Subject 5 - 21Jan2021

Participant ID – Subject # 5

Date of interview: January 21, 2021

Date of Surgery: January 29, 2020

Surgery Performed: L ACL hamstring autograft, PLM, PMM

INTERVIEWER: Can you start by telling me your age

PATIENT: 46

INTERVIEWER: What do you do for work

PATIENT: Nurse

INTERVIEWER: When and how did you injure your knee

PATIENT: To be honest maybe about 10 years ago, learning how to ski. Thought it was a bad twist. Any time try to play basketball with my son, my knee would buckle, and swell. A few months before surgery, I tripped and the pain would not go away. MRI showed a tear.

INTERVIEWER: Any other sports prior to injuring your knee

PATIENT: No – skiing and basketball. Playing basketball until the time of surgery. No skiing after original injury. Recreation basketball. With kids.

INTERVIEWER: When you saw surgeon, did you discuss graft options? Did he explain to you that there are different ways to do the surgery

PATIENT: No, not that I remember.

INTERVIEWER: DO you remember what kinds of graft you had for surgery?

PATIENT: Hamstring

INTERVIEWER: Why did you decided on this graft options

PATIENT: To be honest, I don’t really think he gave me a clear explanation for the plan for surgery

INTERVIEWER: Was it based on his recommendation?

PATIENT: I don’t think I was given any other options. Also said I had some arthritis at the time of surgery and maybe in about 10 years I might need a knee replacement

INTERVIEWER: Are you having any pain in your knee now?

PATIENT: The recovery has been a long difficult time. For one reason, during the pandemic, I wasn’t going to the therapist as much as I would like and the gym was closed. For a few months after surgery, I did some virtual therapy, but it wasn’t the same. Right now it is feeling tight. Its still not there.

INTERVIEWER: Would you describe it in any way as pain in your knee

PATIENT: Ya, it is a 1. It is just tight, a bit

INTERVIEWER: What does that tightness mean to you

PATIENT: For me, I don’t think it is fully recovered yet. There are still things I cannot quite do. I cannot stand for a long time.

INTERVIEWER: When was your surgery

PATIENT: January 29, 2020

INTERVIEWER: When do you feel the tightness in your knee, is it all the time

PATIENT: It pretty much always happens all the time, but it aggravated after standing or walking for a long time.

INTERVIEWER: What makes it better

PATIENT: I guess rest. I mean I have been working almost everyday. Keep it elevated might help a little bit

INTERVIEWER: Does your knee interfere with day to day or sports related activities

PATIENT: Still cant run the way I want to. When I try running on the street, it hurts a little bit more. Bought an elliptical and it doesn’t hurt compared running on road.

INTERVIEWER: Any difficulty with stairs, kneeling, squatting

PATIENT: Not difficulty, but I can feel more pressure when I am kneeling down, or when I walking up stairs

INTERVIEWER: Does you knee affect daily living

PATIENT: No

INTERVIEWER: Does your knee affect ability to return to sports

PATIENT: Because I am still feeling the tightness, I am still hesitant that I might injure it further if I do that. Afraid, in my head, that I might injure it again. It is not quite steady yet. But the thing is, I was walking on the sidewalk the other day, and I tripped and twisted mu ankle, which would have made knee swell before surgery, but it didn’t hurt.

INTERVIEWER: Does knee affect ability to complete job

PATIENT: When I stand for a 12 hour shift, it seems to be too long for me, so I am working in a clinic right now. My job description has changed, but starting to return to modified shift next week

INTERVIEWER: Has your knee affected your quality of life in any way

PATIENT: Still try to have fun, still go for a walk with my kids. Just frustrated that I am not fully recovered, approaching one year since surgery. Work is pushing me to go back to work.

INTERVIEWER: Anything else you want to discuss in regards to your knee

PATIENT: Just frustrating me that it still feels tight and once in a while I have that pain. It is still not steady. When I stand or walk for longer period of time, I have some pain. My knee still hurts me after 2-3 minutes of running

INTERVIEWER: Where in the knee would you say it hurts you?

PATIENT: More just under my patella area

INTERVIEWER: It is pain there with activity

PATIENT: And when I try to test myself, I still feel it after 2-3 minutes. Is the tightness going to go away?


INTERVIEWER: Where is your anterior knee pain (i.e.., behind your kneecap, inside part of knee)?

PATIENT: Inside my kneecap

INTERVIEWER: When does your anterior knee pain bother you (stairs, kneeling, specific activities)?

PATIENT:: Going down the stairs

INTERVIEWER: On a scale of 1-10 (10 being worst pain ever), how would you rate your pain?

PATIENT: 0-1

INTERVIEWER: Is your pain improving, getting worse, or staying the same?

PATIENT: Improving

INTERVIEWER: Do you have any associated symptoms (instability, swelling, numbness etc.)?

PATIENT: Some numbness, but getting less

INTERVIEWER: How would you describe your pain (sharp, stabbing, dull, ache)?

PATIENT: Dull pain once in a while

INTERVIEWER: Does your pain move (radiating pain) ? No

# Subject 6 - 21Jan2021

Participant ID: 6

Date of Interview: January 21, 2021

Age: 45____________

Gender: M

Occupation: Research Associate


Standard Questions for all Patients:


When and how did you injure your knee? This is not completely clear, there were 2 episodes. SOmeimte in summer 2016, playing soccer and jumped up for a ball, but when landed felt something happen to knee. About a year later, I was running and I felt an acute pain in my knee. Not exactly sure when, but had similar complaints in my knee

Did you play sports prior to injuring your knee? Which sports and what level of competition? Soccer and running; That was probably the first time I played soccer in about 30 years. Typically play hockey, tennis, bike, swim. Weekend warrior

When you saw the surgeon, did you talk about the different graft options for ACL reconstruction? I don’t think so. I know we talked about the hmatering, but I am unsure if there were options presented. From discussions before and after, I know we talked about the patella. It is my feeling that the hamstring was presented as the option, but it wasn’t presented as a list of options.

Why did you decide on the one you have? I guess that was what was recommended

If not discussed, what type of ACL reconstruction did you have?

Any pain in your knee? I would say no, it doesn’t feel normal. It is not something that I would consider pain. Feels like muscle stiffnessa round the knee. I wouldn’t consider it pain in the knee


Questions for Someone Who Experiences Anterior Knee Pain (AKP)


What does this knee pain mean to you?

Please describe your experience with anterior knee pain and when it occurs – is it there all the time, are there specific movements that cause pain or worsen the pain?

What relieves the pain?

Does anterior knee pain currently interfere with any day-to-day or sports-related activities? / Is there anything you want to do that your anterior knee pain stops you from doing? (Prompts with below examples, if not mentioned by the patient. Ensure you discuss each category briefly.) No, it feels quitte good. I am just waiting for the green light to do things the way I was doing before. As of right now I am quite happy with the level of activity right now.

Activities: For example, do you have any difficulty you have with going up and down stairs, kneeling, squatting, etc. Please describe? I would say, mabe going up stairs, squyatting. If feels like the area around mu knee is tight, but I wouldn’t say it gives me difficulty doing those things.

ADLs: Does it affect you in domestic work/daily living such as getting dressed, cleaning the house, gardening, driving etc.? Please describe? Does it hinder me in daily activities – no. Able to do all of these activities. Other than feeling that one knee is different, it doesn’t hinder me

Athletics: Has anterior knee pain specifically affected your ability to return to sports? Please describe? Would you be able and willing to get back to playing at the same level as before.

Yes, I think so. Although with getting older I am not sure I can get back to the same level, but I am going to try.

Work: Does the pain affect your ability to complete your job? No

Have you returned to playing (x) sport? What factors influenced your decision to go back/not go back to sports after surgery? – was it due to anterior knee pain? How do you feel about that? Ya, I have been doing some sport. I have not been given the 100% clearance from my PT to returen to sport. I have been plating some low level tennis and I have been skating. I also have a brace from PT before surgery. Antyhgiin where I feel like I might hurt my knee, I wear a brace.

How has the pain affected your quality of life? Fine, great. As I went through the physio process deciding to go forward with the surgery, things deteriorated. My quality of life is better than it was just before the surgery. I can do more things in comparison.

Is there anything else you would like to discuss with respect to anterior knee pain?

I would love to see a little but more integration between the PT and the surgeron. It would be nice if there was just a little more integration and following up on this. I think there should be more communication. I think the PT is experienced with this, and I was seeing this person before surgery and was happy with the way they operated. I think there is a big difference, but at least for me it was much more helpful to interact with someone to follow the prescribed activities. But it was much easier to work with someone. It took me a little while for the PT to setup virtual meets because I had surgery just before the pandemic.


 How much therapy doing now?

See PT every 5 weeks now. I do exercises 5 days a week, foam roller and stretching. 2 days a week, do specific PT related exercises. Another couple of days a week try to do exercises that incorporate specific exercises.


I hope I didn’t call the issues I was having ‘painful’ or ‘weakness'. It’s more of a tight feeling that I don’t associate with a painful feeling. It’s something I notice, but I don’t consider it painful.

Where is your anterior knee pain (ie., behind your kneecap, inside part of knee)? In the muscle behind the inside of my knee. It mostly feels like it’s in the calf, but sometimes it feels like its in the hamstring.


When does your anterior knee pain bother you (stairs, kneeling, specific activities)? Stairs, flexing hamstring motions, when I’m on my feet a lot, like grocery shopping.

On a scale of 1-10 (10 being worst pain ever), how would you rate your pain? As mentioned, I don’t really consider it painful, more of a tight feeling, but if you need a number, then ‘1’.

Is your pain improving, getting worse, or staying the same? moderate improvement

Do you have any associated symptoms (instability, swelling, numbness etc.)? none

How would you describe your pain (sharp, stabbing, dull, ache)? Dull or mild tightness.

Does your pain move (radiating pain) ? No

# Subject 7 - 03Mar2021

Participant ID – Subject #7

Date of interview: March 3, 2021

Date of Surgery: December 9, 2019

Surgery Performed: R ACL Quadriceps


INTERVIEWER: Alright could you start by telling me your age please

PATIENT: I am 44

INTERVIEWER: what do you do for work?

PATIENT: I am a tax accountant

INTERVIEWER: when and how did you injure your knee?

PATIENT: I injured my knee roughly in the summer of 2018 whilst playing ice hockey, recreational ice hockey. So I slammed feet first into the boards traveling at what felt like a great speed and extended my knee, and snapped my ACL

INTERVIEWER: other than hockey did you play any other sports before injuring your knee?

PATIENT: no

INTERVIEWER: when you saw the surgeon did you talk about the different graft options available for ACL reconstruction?

PATIENT: yes, so he mentioned that we could go with a couple of different options. my memory is not perfect about all of them, so he did discuss them, back and that's about the best I can say

INTERVIEWER: why did you decide on the graft that you have?

PATIENT: the recommendation of the surgeon. I also think that he there may have been some change in in the decision making process after the surgery began I'm not sure about that

INTERVIEWER: what do you mean by that?

PATIENT: never mind, to be honest my memory on the particular options is very poor

INTERVIEWER: what type of graph did you have?

PATIENT: I'm supposed to know the answer to that aren't I?

INTERVIEWER: where is your biggest incision is it above the kneecap or below the kneecap?

PATIENT: Above the knee cap

INTERVIEWER: Ok, that's what I thought I just wanted to confirm, you had a quadriceps graft. Are you having any pain in your knee right now?

PATIENT: it's still not back to 100%, but I have full range of mobility, but there is no pain

INTERVIEWER: no pain, just doesn't feel 100%?

PATIENT: correct, and as a result of COVID, my physio appointments were quickly cancelled at probably just the wrong time

INTERVIEWER: understood we'll talk about some specific type activities now, but do you have any problems going up or down stairs?

PATIENT: no

INTERVIEWER: kneeling or squatting?

PATIENT: certainly my agility in that department is less, it might be psychological too just because I'm very conscious I don't want to get injured

INTERVIEWER: Has your surgery and recovery affected your daily life such as cooking cleaning or getting dressed?

PATIENT: over what time frame? I mean obviously it affected my life in the few months following, but I'm not quite sure what time frame your question is encompassing

INTERVIEWER: so more recently now that you've kind of had a little over a year to recover?

PATIENT: Zero, absolutely. If the world were different I would be back on the ice playing hockey right now

INTERVIEWER: does it prevent you from doing your job?

PATIENT: no, my my job involves me sitting at a desk for 12 hours a day which is probably what led to the injury in the first place

INTERVIEWER: I think you've you touched on this but would you feel comfortable returning to play hockey now?

PATIENT: yes, but you know, with some reservation I'd be playing with a knee brace and I'd be a lot more mindful of injury. But yes I would like to go back, I will go back, I'm going to

INTERVIEWER: when was the last time you were on the ice do you remember?

PATIENT: that would've been, the surgery was December, the last time I was on the ice was May of 2020

INTERVIEWER: ok, so you haven’t been able to get back on the ice recently?

PATIENT: all the leagues were cancelled, which is probably blessing for me. I really wanted to get back just because I don't do have any other sport and I wanted to get back and start doing fitness again. I might have gone back too soon if leagues were not cancelled last season, last winter

INTERVIEWER: is there anything you want to do that your knee stops you from doing?

PATIENT: jogging is a very high impact thing and if I go jogging now my knee swells up so I would like to do running but mine is preventing me from doing that, trail running kinda cross country stuff. I go for a walk every day but there's no way I can only reach a certain pace before my knee starts to hurt

INTERVIEWER: when it hurts where does it bother you ?

PATIENT: just above the knee cap

INTERVIEWER: is it the only time it bothers you when you are walking the dog?

PATIENT: if I try running, that’s when it bothers me. Like I said there's a certain stiffness. If I was doing proper physio to build back up the strength that probably wouldn't bother me as much. Stiffness and like a bit of weakness it does it's not 100% if I'm just sitting here no problem

INTERVIEWER: how do you feel your quality of life is following surgery?

PATIENT: excellent, I think the surgery went very well and I'm glad that I did it. Prior to the surgery it would be OK and every now and then I would be on a wet floor and I would slip and I tweaked my knee and it would swell up for two weeks, and wouldn’t be able to walk. just avoiding the problems I was having before by addressing them has been pretty great improvement not my quality of life

INTERVIEWER: OK good and is there anything else you'd like to discuss with respect your surgery or recovery?

PATIENT: in my view it was a raging success all the way across the board

INTERVIEWER: that's all I have for now I'm gonna stop recording here

# Subject 8 - 03Mar2021

Participant ID: 8

Date of Interview: March 3, 2021

Age: 19

Gender: M

Occupation: Grocery Store, part time

Standard Questions for all Patients:

When and how did you injure your knee? I tore it coming on 2 years now, playing basketball. I was just getting out of school, playing basketball with some friends and I landed weird. Then it took 6-8 months to get it fixed.

Did you play sports prior to injuring your knee? Which sports and what level of competition? Used to play soccer, pretty much it. Recreational, house league on the side. Also played basketball

When you saw the surgeon, did you talk about the different graft options for ACL reconstruction? Yes. He was just explaining where the grafts came from.

Why did you decide on the one you have? I want to be an electrician, so he was saying the quadriceps is stronger in the long term and it will be easier to crouch, it will not be as fatigued as easily.

If not discussed, what type of ACL reconstruction did you have? Quadriceps

Pain in the knee? Its odd. I have been snowboarding since then, and it has held up just fine. But at the same time, sometimes when I am just walking my dog, every so of ten it pops out, it feels like, and I have to pull it back. Like to stretch the quad, bring your heel to your butt, and pop it back and it goes away. Also, I get some weird fatigue, it feels like 2 major tendons, and one of them on the inside, every once in a while, it just gets fatigued and it hurts. When I went snowboarding I came back and I was fine and then the next day it was damp out and it hurt.
Questions for Someone Who Experiences Anterior Knee Pain (AKP)

What does this knee pain mean to you? Its like a pain that is constantly there. But it fades away after a few minutes. It is not a sharp pain, occasionally it is sharp, but it does not stay there. There are 2 things that are painful, one is the popping and the other is on the inside, on the hamstring.


Please describe your experience with anterior knee pain and when it occurs – is it there all the time, are there specific movements that cause pain or worsen the pain?

Sitting in one spot and then having to squat up and down, like I am at work and if I have to squat down low, it makes it worse. Rest makes it better. And I have been working out on it and it feels fine when it is under weight, but repetitive movement stresses it and makes it worse.

What relieves the pain? Rest

Does anterior knee pain currently interfere with any day-to-day or sports-related activities? No

Is there anything you want to do that your anterior knee pain stops you from doing? (Prompts with below examples, if not mentioned by the patient. Ensure you discuss each category briefly.)

Activities: For example, do you have any difficulty you have with going up and down stairs, kneeling, squatting, etc. Please describe? No. Sometimes I can walk and it is fine and sometimes it gives me problems.

ADLs: Does it affect you in domestic work/daily living such as getting dressed, cleaning the house, gardening, driving etc.? Please describe? No problems there

Athletics: Has anterior knee pain specifically affected your ability to return to sports? Please describe? I haven’t really gone out, I have played a little bit of soccer, but I haven’t really gotten a chance with this whole corona virus.

Work: Does the pain affect your ability to complete your job? If it does hurt, I just need to take it easy for a moment. Or if it just pops, I need to take a minute, there is always something else I can be doing

Have you returned to playing (x) sport? What factors influenced your decision to go back/not go back to sports after surgery? – was it due to anterior knee pain? How do you feel about that? I have returned to snowboarding.

How has the pain affected your quality of life? No, ever since the brace came off it has been great

Is there anything else you would like to discuss with respect to anterior knee pain? I don’t think I got to go to my 8 month follow up appointment, is there another chance for that to happen?

# Subject 9 - 03Mar2021

Participant ID: 9

Date of Interview: March 3, 2021

Age: 32

Gender: M

Occupation: Runs Internet Business

Right knee ACL

Standard Questions for all Patients:

When and how did you injure your knee? Initially I was playing volleyball, it was the first time in 5 years. I wasn’t doing any sports in those 5 years, just lifting weights. First game back, I jumped to spike a ball and just landing, the impact, I dislocated my knee and tore my ACL. I didn’t even go to the doctor or antthuing, a month later I was feeling better but I knew something was wrong with it. And then I was a t the cottage and I stepped off a paddleboard and it dislocated again, so it was back to recovery, and this time it was worse. The final blow came when I was playing bubble soccer and I got hit by somebody and I absorbed the impact and it was significantly worse and I couldn’t even walk without a brace. SO I finally went to the doctor and got prescribed surgery. I believe this was in May 2019. Surgery was November 3, 2019

Did you play sports prior to injuring your knee? Which sports and what level of competition? I never played soccer, the time that I discloated it I was at a birthday party. I have played volleyball. An avid weight lifter for years. Jogging too.

When you saw the surgeon, did you talk about the different graft options for ACL reconstruction? No, I think he may have told me what graft we will be doing but never talked about the options.

Why did you decide on the one you have?

If not discussed, what type of ACL reconstruction did you have? Quadriceps tendon.

Pain in the knee right now?

It’s complicated. I have a recurrent tracking problem in my knee right now. It will especially happen if I am doing any high impact activity, like running or jumping. It may also happen depending on how intense the activity. These can impact the tracking and pain. Can get a really intense shooting pain. I will have to straighten my leg and re-adjust.

Location of the pain when it happens? Outside of my kneecap, almost like where the IT band area. There is a lot of pressure there. Also, on the inside of the kneecap is where I will get the intense shooting pain. If I put my finger on the center of the kneecap and move my finger 2 inches inwards, that is where I will feel it.

Questions for Someone Who Experiences Anterior Knee Pain (AKP)

What does this knee pain mean to you? Its extremely debilitating. Any sort of physical activity I do now revolves around making adjustments to prevent the pain from happening. It is also the focus on physiotherapy to make it better. There is also a ton of activities and sports that I avoid in order to not make it worse.

Please describe your experience with anterior knee pain and when it occurs – is it there all the time, are there specific movements that cause pain or worsen the pain?

What relieves the pain? Its definitely improved the more muscle I build on my leg, so the thing that relieves it the most is just doing physio. Yes, it is still getting better. For a long time my leg was so weak, it was very hard to make progress because even a small amount of activity would give me tracking problems for a full week after physio. I am at the point where I am able to more activity. I am able to build muscle a lot faster now and It is really starting to improve.

Does anterior knee pain currently interfere with any day-to-day or sports-related activities? / Is there anything you want to do that your anterior knee pain stops you from doing? (Prompts with below examples, if not mentioned by the patient. Ensure you discuss each category briefly.)

I hacvent been able to go biking, skiing or snowboarding. Even basic things like skipping rope.

Activities: For example, do you have any difficulty you have with going up and down stairs, kneeling, squatting, etc. Please describe?
I do feel weakness in the right knee that I don’t feel in the left knee. It improves the more muscle I can feel on my right leg. It is a very noticeable difference, stepping up with the right leg, and the weakness.

ADLs: Does it affect you in domestic work/daily living such as getting dressed, cleaning the house, gardening, driving etc.? Please describe?

The tracking pain definitely will, like if I just doing random stuff around the house, going up the stairs, doing laundry, you know the tracking problem is pretty debilitating.

Athletics: Has anterior knee pain specifically affected your ability to return to sports? Please describe?
Yes, for sure. The only physical activity I do now is specifically for building muscle in my leg. I am also very apprehensive about doing it again. I don’t know if I will ever feel confident again, but I feel like a long way out before I feel confident doing spiorts and stuff.

Work: Does the pain affect your ability to complete your job?

No, I am lucky in that sense because I am working on a computer.

Have you returned to playing (x) sport? What factors influenced your decision to go back/not go back to sports after surgery? – was it due to anterior knee pain? How do you feel about that?

How has the pain affected your quality of life? The whole experience has been very difficult. You know, looking back when I got the surgery, I came in naïve. I was thinking of the athletes that recover in 4-6 months, and thinking of myself as an athlete I though this would be a piece of cake. After the surgery what was surprising for me, the problems that I felt went beyond my knee. I developed some nerve pain throughout my lower body. I developed back pain and shoulder. I feel like my body is not the same as it was before the surgery. I cant recover from injury as quickly. All my muscles are very sensitive so the last year has not only been rehabbing my knee but trying to figure out what is wrong with my body. I was in the gym 5 days a week prior to my surgery and after surgery, lifting weights was out of the question for months because of my back and my shoulder. I have completely reimagined how I take care of my body. It has been a long recoevery.

Is there anything else you would like to discuss with respect to anterior knee pain?

I feel what happened in my case, because I had a difficult recovery. I feel like there was a communication breakdown early on after surgery that set me off on a path to a poor recovery. Going into surgery I was naïve about how important PT was in your recovery. I was prescribed a physio, but after a month I felt like I wasn’t getting great treatment, and I was paying a lot of money. The physio was giving me exercises straight out of a book, so I went to another physio and the same thing. It turned out I had a tracking issue and not really understood what was going on with the tracking issue. So I was naice, not understanding the importance of physio. I was not made a aware by the surgeon before surgery how important physio is in the recovery. I went through 4 physiotherapists.

# Subject 10 - 05Mar2021

Participant ID: 10

Date of Interview: 05Mar2021

Age: 32

Gender: Male

Occupation: Realtor, payroll accountant

Standard Questions for all Patients:

When and how did you injure your knee?

I tore it playing baseball, running into second base. Knee on knee collision, may have injured prior to impact.

Did you play sports prior to injuring your knee? Which sports and what level of competition?

Baseball, pretty much all of them, football, basketball, hockey, tennis, squash, run a little bit. Competitive mens league caliber, just like men’s league. Recreational

When you saw the surgeon, did you talk about the different graft options for ACL reconstruction?

Yep, we went over all of the options and some of the different pros and cons of each. We settled on the patella because it had a good success rate. A little longer recovery, but it is worth it in my mind. Biggest scar is above the kneecap

Why did you decide on the one you have?

If not discussed, what type of ACL reconstruction did you have? Quadriceps.

Any pain in the knee? Every now and then, it is not 100% id say, it’s a little creaky. Pain is right in the gap, just right of the kneecap (left knee). Also a little bit of a tingling feeling, like if I go down on my knee for more than 30 seconds I feel it, and there is some tingling too.

Questions for Someone Who Experiences Anterior Knee Pain (AKP)

What does this knee pain mean to you?

Its tolerable, manageable. Prefer it be gone altogether. It is not prohibitive for day to day things. It is hard to get dfown on my knees, it made being on my knees difficult.

Please describe your experience with anterior knee pain and when it occurs – is it there all the time, are there specific movements that cause pain or worsen the pain?

I have been fairly inactive recently, so I have not been playing any sports. So I don’t know how the knee is going to respond when I test it out.

What relieves the pain?

Just getting off my knees. Releasing the direct contact.

Does anterior knee pain currently interfere with any day-to-day or sports-related activities? / Is there anything you want to do that your anterior knee pain stops you from doing? (Prompts with below examples, if not mentioned by the patient. Ensure you discuss each category briefly.)

Activities: For example, do you have any difficulty you have with going up and down stairs, kneeling, squatting, etc. Please describe?

A little bit, not difficulty, I can do it, but I feel it still. Issues with kneeling, that is when it bothers me. Squatting is bothersome as well. But I haven’t necessarily done the appropriate strength training because of COVID, not able to go to physio or the gym and stuff.

ADLs: Does it affect you in domestic work/daily living such as getting dressed, cleaning the house, gardening, driving etc.? Please describe?

No, all that is generally pretty good. Driving if it is for long periods, the knee gets stiff and sore because it has been bent for a long time.

Athletics: Has anterior knee pain specifically affected your ability to return to sports? Please describe?

No, I played golf through the summer, and that was fine. The range of motion is limiting, but it is not the end of the world. I haven’t really jumped back in because I was told to give it at least a year. I have skated a couple of times, but that is about it.

Work: Does the pain affect your ability to complete your job?

No, I work in an office

Have you returned to playing (x) sport? What factors influenced your decision to go back/not go back to sports after surgery? – was it due to anterior knee pain? How do you feel about that?
I am hopeful I can return to play sports. If I just ease my way back in, I think it will be ok. It is not at the point where I don’t notice it at all, but because I notice it, it is not 100%

How has the pain affected your quality of life?

Not significantly, it is less than ideal, but it is not prohibitive

Is there anything else you would like to discuss with respect to anterior knee pain?

Nothing specifically, as it pertains to the survey.

# Subject 11 - 05 Mar2021

Participant ID: 11

Date of Interview: 05Mar2021

Age: 45

Gender: M

Occupation: Work for an electric skateboard company

Standard Questions for all Patients:

When and how did you injure your knee?

I think my surgery was last February, so I think the injury occurred a year to 6 months before surgery. This is due to the extent of the injury to the knee, but I was walking around without issue, but only had minor issues with the mechanics of the knee. It was only when I went to get the knee checked out that I realized that I tore the ACL. So, I was at an indoor skate park and I performed a manouver, and when I was in the air, one of my legs was tucked under my leg and when I landed, I landed with my leg under me. I heard a pop, I am not sure if I heard it or if I felt it, but there was a pop. It was instant pain. I assume that the injury happened at that time. I couldn’t stand. This was just me skateboarding on my own .

Did you play sports prior to injuring your knee? Which sports and what level of competition?

I am also active in 2 martial arts. This is just lifestyle. I don’t compete, it is just something I do

When you saw the surgeon, did you talk about the different graft options for ACL reconstruction?

Yes, of course I was very ignorant. But I was told the options and the pros and cons. The recimmnedations based on my specific case. SO I was very clear on my options at that time.

Why did you decide on the one you have? I can’t remember exactly because it ewas a long time ago, but it was the most recommended one.

If not discussed, what type of ACL reconstruction did you have?

It was explained to me that it was an autograft from my hamstring.

Any pain in the knee? So, at this moment, generally no. But there are certain positions where the knee is a little but sore. I have not regained full flexion yet, but one of my concerns is that I want my foot to reach my buttocks. I do experience some pain as I flex the knee more. But in daily activities, generally no pain. The discomfort is really in the knee joint, right below the knee cap. Also had surgery on meniscus, and that is where I feel the pain.

Questions for Someone Who Experiences Anterior Knee Pain (AKP)

What does this knee pain mean to you?

It feels like there might be something mechanical there that is limiting the movement. As I get closer to a deep squat, I experience some resistance. I have been told that it is some scar tissue or inflammation that I need to work through.

Please describe your experience with anterior knee pain and when it occurs – is it there all the time, are there specific movements that cause pain or worsen the pain?

Its mainly the squatting, just the squatting. There was a recent event where I fell while skateboarding, legs were in the air and it caused a little bit of pain, but this went away.

What relieves the pain?

Stopping squatting. If I feel that pain, either I will not continue to go into that squat or straighten the leg to relieve it.

Does anterior knee pain currently interfere with any day-to-day or sports-related activities? / Is there anything you want to do that your anterior knee pain stops you from doing? (Prompts with below examples, if not mentioned by the patient. Ensure you discuss each category briefly.)

Activities: For example, do you have any difficulty you have with going up and down stairs, kneeling, squatting, etc. Please describe? No issues with stairs. Pain with kneeling. That is another issue. Just a lifestyle thing, my family is Japanese and we traditionally sit in a kneeling position, so I will do it as long as I can

ADLs: Does it affect you in domestic work/daily living such as getting dressed, cleaning the house, gardening, driving etc.? Please describe?

No, not at all

Athletics: Has anterior knee pain specifically affected your ability to return to sports? Please describe?

It has, but only because I am aware that my knee is not 100% strong, and it doesn’t have the confidence that the leg is ready for it.

Work: Does the pain affect your ability to complete your job?

No

Have you returned to playing (x) sport? What factors influenced your decision to go back/not go back to sports after surgery? – was it due to anterior knee pain? How do you feel about that?

I have basically returned to all the sports I am involved with, but it is in a reduced capacity.

How has the pain affected your quality of life?

I would say that it has just reduced the fun factor, when it comes to sport I do it goes against my nature to be reserved. But I am definitely conscious of the that.

Is there anything else you would like to discuss with respect to anterior knee pain?

My knee surgery happened last February, and shortly thereafter the pandemic hit so I basically did not go and see a physiotherapist. Just given the circumstances. So I know for certain that I did not have a chance to re-train the muscles that I would have otherwise.

# Subject 12 - 10Mar2021

Participant ID: 12

Date of Interview: 10Mar2021

Age: 34

Gender: M

Occupation: COnstruction Project Manager

Standard Questions for all Patients:

When and how did you injure your knee?

I first injured it about 4 years ago playing ultimate disc on a turf field, just kind of made a funny movement and my ankle rolled and I injured my knee.

Did you play sports prior to injuring your knee? Which sports and what level of competition?

Yes, primarily also played hockey, little bit of softball. Very casual, amateur.

When you saw the surgeon, did you talk about the different graft options for ACL reconstruction?

We did.

Why did you decide on the one you have? We mostly talked about quadriceps tendon, and patellar tendon I believe. The surgeon recommended the quadriceps tendon as one he has had success with in athletes. SO I took that information and did my own research and found there is reasonable success with both options of grafts, so I was happy to go with the surgeons recommendation.

If not discussed, what type of ACL reconstruction did you have?

Quadriceps

Surgery was December 10, 2019

Pain in the knee? Typically no. Sometimes, in certain positions I will get some soreness and I need to stretch it out.

Questions for Someone Who Experiences Anterior Knee Pain (AKP)

What does this knee pain mean to you?

Please describe your experience with anterior knee pain and when it occurs – is it there all the time, are there specific movements that cause pain or worsen the pain

What relieves the pain?

Does anterior knee pain currently interfere with any day-to-day or sports-related activities? Is there anything you want to do that your anterior knee pain stops you from doing? (Prompts with below examples, if not mentioned by the patient. Ensure you discuss each category briefly.)

Activities: For example, do you have any difficulty you have with going up and down stairs, kneeling, squatting, etc. Please describe? No issues with stairs. It still more painful than my other knee when kneeling on hard surfaces. Point of contact is painful with kneeling, where my knee making contact.

ADLs: Does it affect you in domestic work/daily living such as getting dressed, cleaning the house, gardening, driving etc.? Please describe?

No

Athletics: Has anterior knee pain specifically affected your ability to return to sports? Please describe?

No, small caveat I really haven’t had the opportunity, otherwise I would have been looking to get back to sports at the end of the last year.

Work: Does the pain affect your ability to complete your job? No

Have you returned to playing (x) sport? What factors influenced your decision to go back/not go back to sports after surgery? – was it due to anterior knee pain? How do you feel about that?

I don’t have 100% confidence, before I know I am going to go back, I would like to build the muscle. Would like to make sure I have done more for the muscles in my leg.

How has the pain affected your quality of life?

Has not affected


Is there anything else you would like to discuss with respect to anterior knee pain?

I wasn’t able to go through all of my postoperative checkups, so I would like be able to do that and I might have more confidence returning to sport.

# Subject 13 - 16Mar2021

Participant ID: 13

Date of Interview: 16Mar2021

Age: 18

Gender: F

Occupation: Student

Standard Questions for all Patients:

When and how did you injure your knee?

I injured my knee August 2018 playing soccer. I got tripped and twisted my knee. I wasn’t sure it was an ACL tear right away. I didn’t get it looked at right away. Then a couple of months later I was playing lacrosse and I hurt it again , got an MRI and ACL was torn. At that point I was playing a season of hockey and I had playing without an ACL.

Did you play sports prior to injuring your knee? Which sports and what level of competition?

Soccer, lacrosse and hockey. Soccer was just house league for fun. School lacrosse. Hockey, playing junior in the PWHL.

When you saw the surgeon, did you talk about the different graft options for ACL reconstruction?

Yes, I did. He gave me 3 options, hamstring, patellar, and cadaver.

Why did you decide on the one you have?

I did some research and thought the patella would heal better because it was my own tissue. Did not want to have to recover hamstring after surgery .

If not discussed, what type of ACL reconstruction did you have? Patellar tendon

Surgery was February 20, 2020

Pain in the knee? I have a little bit. Sometimes after a long workour or after a game, I will have some pain. It is almost fatigue pain. I would say the pain is around the patellar tendon, or inside, just below the kneecap.

Questions for Someone Who Experiences Anterior Knee Pain (AKP)

What does this knee pain mean to you?

I think it means that I have fully recovered and its just something that will always be there, and it is just a little bit of tendonitis. It is a sign of hard work and it will pass, and it does.

Please describe your experience with anterior knee pain and when it occurs – is it there all the time, are there specific movements that cause pain or worsen the pain?

So it happened usually after long periods of working out, or after a hockey game. If I am really pushing my knee, I will feel it afterwards.

What relieves the pain?

Ice. I stretch it, walk, focus on stretching it out. It usually comes back pretty quickly.

Does anterior knee pain currently interfere with any day-to-day or sports-related activities? / Is there anything you want to do that your anterior knee pain stops you from doing? (Prompts with below examples, if not mentioned by the patient. Ensure you discuss each category briefly.) Nope, I am able to do everything. My trainer doesn’t allow to play any field sports. I wear my brace sometimes, and I am a little bit more cautious. Sometimes I am unable to run, but I find myself limping.

Activities: For example, do you have any difficulty you have with going up and down stairs, kneeling, squatting, etc. Please describe? No issues with stairs. Kneeling on that knee on a hard surface, it hurts. Squatting is fine.

ADLs: Does it affect you in domestic work/daily living such as getting dressed, cleaning the house, gardening, driving etc.? Please describe?

No, none of those.

Athletics: Has anterior knee pain specifically affected your ability to return to sports? Please describe? Nope.

Work: Does the pain affect your ability to complete your job? Nope.

Have you returned to playing (x) sport? What factors influenced your decision to go back/not go back to sports after surgery? – was it due to anterior knee pain? How do you feel about that?

Yes I have. I returned to play 6-7 months after my surgery.

How has the pain affected your quality of life?

No it has not

Is there anything else you would like to discuss with respect to anterior knee pain?

Nope, everything went well and everything is good now. Feels a lot better now compared to before surgery because I had it torn for 2 years before surgery. No instability.

# Subject 14 - 31Mar2021

Participant ID: ______14___________

Date of Interview: _____March 31, 2021_____________

Age: ____________ 38

Gender: _____M_____

Occupation: _____________________________________ Education - teacher


Standard Questions for all Patients:

When and how did you injure your knee? I tore my ACL in 2005. I was doing vertical jump testing, landed on one leg and tore my ACL

Did you play sports prior to injuring your knee? Which sports and what level of competition? Playing hockey, playing 5-6 days per week – NCAA; lacrosse – recreation

When you saw the surgeon, did you talk about the different graft options for ACL reconstruction? Believe so – 2 options hamstring vs cadaver

Why did you decide on the one you have? own body, no chance of rejection,

If not discussed, what type of ACL reconstruction did you have? Hamstring – Feb

Pain in the knee? back left (posterolateral); a little bit of pain in the front of the knee; doesn’t happen very often

Questions for Someone Who Experiences Anterior Knee Pain (AKP)

What does this knee pain mean to you? Not enjoyable; it (pain) has gotten worse as the year gone by; it is the same or slowly progressing in pain

Please describe your experience with anterior knee pain and when it occurs – is it there all the time, are there specific movements that cause pain or worsen the pain? Walks, physical activity, will feel afterwards, aching type pain; worse with activity

What relieves the pain? Rest, ice packs, TENS machine

Does anterior knee pain currently interfere with any day-to-day or sports-related activities? No running sports for many years

Is there anything you want to do that your anterior knee pain stops you from doing? (Prompts with below examples, if not mentioned by the patient. Ensure you discuss each category briefly.) Unable to take longer walks, limited to 4-5 km, can still bike, cannot play with kids, and own physical health as a result of pain

Activities: For example, do you have any difficulty you have with going up and down stairs, kneeling, squatting, etc. Please describe? Pain with Kneeling, no issues with stairs

ADLs: Does it affect you in domestic work/daily living such as getting dressed, cleaning the house, gardening, driving etc.? Please describe? Does not affect

Athletics: Has anterior knee pain specifically affected your ability to return to sports? Please describe? Not anterior knee pain; unable to play running sports

Work: Does the pain affect your ability to complete your job? No

Have you returned to playing (x) sport? What factors influenced your decision to go back/not go back to sports after surgery? – was it due to anterior knee pain? How do you feel about that? Was able to play hockey with knee injury; back on the ice, playing and doing some developmental activities

How has the pain affected your quality of life? Mental health aspect, thinking about future; will it get better or worse, will it progress, other surgeries needed?

Day-to-day stuff, do I need to rest, can we go shopping for extended period of time etc

Is there anything else you would like to discuss with respect to anterior knee pain? No

Where is your anterior knee pain (ie., behind your kneecap, inside part of knee)? back left of knee (posterolateral)

When does your anterior knee pain bother you (stairs, kneeling, specific activities)? walking mostly

On a scale of 1-10 (10 being worst pain ever), how would you rate your pain? 5

Is your pain improving, getting worse, or staying the same? not getting better, probably a slow decline

Do you have any associated symptoms (instability, swelling, numbness etc.)? no instability, don’t notice any swelling

How would you describe your pain (sharp, stabbing, dull, ache)? can be sharpish

Does your pain move (radiating pain) ? No

# Subject 15 - 13Jul2021

Participant ID: __________15

Date of Interview: ____13Jul2021______________

Age: _____29_______

Gender: _____M_____

Occupation: ______architectural drafting_______________________________

Standard Questions for all Patients:

When and how did you injure your knee?

Several injuries, first twisted knee playing soccer and walked if off. Second injury jumping of a ledge, landed awkward. Third, fell of bike.

Did you play sports prior to injuring your knee? Which sports and what level of competition?

Soccer – recreational; lots of cycling

When you saw the surgeon, did you talk about the different graft options for ACL reconstruction? Yes, 2 types – patellar tendon and hamstring

Why did you decide on the one you have? Surgeon recommendation

If not discussed, what type of ACL reconstruction did you have? Most applicable for his level of activity

Any pain in the front of the knee?

If overexert knee, stretching sensation. Posterior knee. Rarely pain in front of knee. Some popping

Questions for Someone Who Experiences Anterior Knee Pain (AKP)

What does this knee pain mean to you? Take activities slower, cannot go on a walk for longer than 10 mins. Can ride bike. Can drive. Still feels like he is recovering.

Please describe your experience with anterior knee pain and when it occurs – is it there all the time, are there specific movements that cause pain or worsen the pain?

Running/sprinting and long hikes with elevation. Does not trust knee yet for explosive activities. Feels as though he needs to continue strengthening. Unable to kick a ball. Too much impact.

What relieves the pain? Rest. Laying down. Elevating leg. Finding comfortable sleeping position was one of biggest challenges

Does anterior knee pain currently interfere with any day-to-day or sports-related activities? Is there anything you want to do that your anterior knee pain stops you from doing? (Prompts with below examples, if not mentioned by the patient. Ensure you discuss each category briefly.)

Activities: For example, do you have any difficulty you have with going up and down stairs, kneeling, squatting, etc. Please describe? Some difficulty going down stairs. Still has some instability/shaking. No pain with kneeling.

ADLs: Does it affect you in domestic work/daily living such as getting dressed, cleaning the house, gardening, driving etc.? Please describe? No issues

Athletics: Has anterior knee pain specifically affected your ability to return to sports? Please describe? Cannot kick a ball

Work: Does the pain affect your ability to complete your job? No

Have you returned to playing (x) sport? What factors influenced your decision to go back/not go back to sports after surgery? – was it due to anterior knee pain? How do you feel about that? No – does not feel like he could sprint or kick a ball explosively. Able to cycle daily. Uses it as a mode of transportation

How has the pain affected your quality of life? Improved significantly after the first part of recovery. Last 6 months gotten better

Is there anything else you would like to discuss with respect to anterior knee pain?

Continue to work on exercises/activities

Cycling works the best for strengthening.

Where is your anterior knee pain (ie., behind your kneecap, inside part of knee)?

Slight pain, posterior

When does your anterior knee pain bother you (stairs, kneeling, specific activities)?

Going down stairs
On a scale of 1-10 (10 being worst pain ever), how would you rate your pain?

2-3

Is your pain improving, getting worse, or staying the same?

Continued gradual progress, helps keep motivated

Do you have any associated symptoms (instability, swelling, numbness etc.)?

Feels he still has some instability, some shaking with activity

How would you describe your pain (sharp, stabbing, dull, ache)?

Dull pain, slight pain

Does your pain move (radiating pain) ?

No radiating pains
